# Racial Concordance on Healthcare Use within Hispanic Population Subgroups

**DOI:** 10.1007/s40615-023-01700-8

**Published:** 2023-07-21

**Authors:** Alyson Ma, Jason Campbell, Alison Sanchez, Steven Sumner, Mindy Ma

**Affiliations:** 1 Knauss School of Business, Department of Economics, University of San Diego, 5998 Alcalá Park, San Diego, CA 92110 USA; 2 Department of Psychology & Neuroscience, Nova Southeastern University, Fort Lauderdale, FL USA

**Keywords:** Concordance, Utilization, Racial disparities, Hispanic, Latino

## Abstract

**Objective:**

To examine the association of patient-provider racial and ethnic concordance on healthcare use within Hispanic ethnic subgroups.

**Methods:**

We estimate multivariate probit models using data from the Medical Expenditure Panel Survey, the only national data source measuring how patients use and pay for medical care, health insurance, and out-of-pocket spending. We collect and utilize data on preventive care visits, visits for new health problems, and visits for ongoing health problems from survey years 2007–2017 to measure health outcomes. Additionally, we include data on race and ethnicity concordance, non-health-related socioeconomic and demographic factors, health-related characteristics, provider communication characteristics, and provider location characteristics in the analysis. The sample includes 59,158 observations: 74.3% identified as Mexican, 10.6% identified as Puerto Rican, 5.1% identified as Cuban, 4.8% identified as Dominican, and 5.2% classified in the survey as Other Hispanics. Foreign-born respondents comprised 56% of the sample. A total of 8% (4678) of cases in the sample involved Hispanic provider-patient concordance.

**Results:**

Hispanic patient-provider concordance is statistically significant and positively associated with higher probabilities of seeking preventive care (coef=.211, *P*<.001), seeking care for a new problem (coef=.208, *P*<.001), and seeking care for an ongoing problem (coef=.208, *P*<.001). We also find that the association is not equal across the Hispanic subgroups. The association is lowest for Mexicans in preventive care (coef=.165, *P*<.001) and new problems (coef=.165, *P*<.001) and highest for Cubans in preventive care (coef=.256, *P*<.001) and ongoing problems (coef=.284, *P*<.001). Results are robust to the interaction of the Hispanic patient-provider concordance for the Hispanic patient categories and being foreign-born.

**Conclusions:**

In summary, racial disparities were observed in health utilization within Hispanic subgroups. While Hispanic patient-provider concordance is statistically significant in associating with healthcare utilization, the findings indicate that this association varies across Hispanic subpopulations. The observations suggest the importance of disaggregating Hispanic racial and ethnic categories into more similar cultural or origin groups. Linked with the existence of significant differences in mortality and other health outcomes across Hispanic subgroups, our results have implications for the design of community health promotion activities which should take these differences into account. Studies or community health programs which utilize generalized findings about Hispanic populations overlook differences across subgroups which may be crucial in promoting healthcare utilization.

## Introduction

Racial and ethnic disparities in insurance coverage, access to care, and healthcare quality have received much attention in recent years [1, 2]. Yet, despite growing recognition and increased attention to mitigating these disparities, they have persisted and, in some cases, widened [2]. An extensive literature examines the relationship between a range of measures of access, insurance coverage, utilization of healthcare services, health outcomes, and discrimination. A well-established finding is that, regardless of their racial or ethnic identity, Hispanic patients continue to have lower rates of health utilization [2–6], indicating that despite improvements over time in access and coverage Hispanic patients are still less likely to engage healthcare services. Lower rates of utilization are also accompanied by lower rates of adherence to prescribed medications and medical interventions [7, 8] and worse health outcomes as well as lower life expectancy [7, 8]. Racial and ethnic discrimination in healthcare settings has also been shown to have an impact on health utilization. Previous work has demonstrated that Hispanic patients receive differential treatment and/or advice from their physicians [9], are more likely to report experiencing both acute and chronic discrimination than their non-Hispanic White peers [10], and are more likely to delay medical care in the face of discrimination [11].

The Affordable Care Act (ACA) improved health outcomes among the Hispanic population—such as a reduction in the probability of poor health status and increases in hypertension control—by expanding access to insurance, improving consumer protections, and reducing financial barriers to utilization [6, 12–14]. However, significant health inequalities between Non-Hispanic White and Hispanic patients (regardless of racial identity) persist, suggesting that poor access and financial barriers are not the only impediments to effective and equitable care [12]. One factor often hypothesized to reduce disparities and improve patient outcomes is shared racial and ethnic identity between patient and provider, known as patient–provider racial and ethnic concordance, particularly for Hispanic patients [3, 12]. This strand of literature argues that physicians from underrepresented groups may have cultural insights, knowledge, and experience that improve patient-provider communication, satisfaction, and health outcomes for minority patients [15, 16].

Language concordance also plays a role in healthcare utilization. A recent randomized clinical trial found that patients rated direct-Spanish care (i.e., having a Spanish-speaking provider) more highly in perceived opportunity to disclose concerns, physician empathy, confidence in physician abilities, and general satisfaction with their physician. Additionally, patients in direct-Spanish care were more likely to initiate unprompted speech and asked their providers more questions [17]. This supports the hypothesis that patient-provider racial and ethnic concordance is a proxy for improved communication between patient and provider, which has been shown to improve health outcomes for minority individuals [18, 19].

The association of racial and ethnic concordance on improved health outcome is correlated by the degree of acculturation [15]. The increasingly diverse demographic makeup of recent immigrant groups contributes to the increasing complexity and diversity of racial and ethnic groups within the USA. As a reflection of the increasing diversity within racial and ethnic groups, recent studies have stressed the importance of not only focusing on health disparities between racial and ethnic groups but also on health disparities within racial and ethnic groups [4, 20]. Focusing on broad racial and ethnic group comparisons ignores the considerable within-group variation. It has been found that diverse ancestry and migration experiences are associated with distinctive vulnerabilities that translate into health inequities [21]. This heterogeneity may account for the mixed findings regarding Hispanics and the association with racial and ethnic concordance. While multiple studies have shown that racial and ethnic patient-provider concordance matters for Hispanic patient visits to their usual source of care (USC) [3, 6], other studies have found that in some cases racial and ethnic concordance may not predict Hispanic patient satisfaction [22–26].

Commonalities help form a cultural identity shared across individuals identifying as Hispanics [12], but many diverse populations fall within this broad ethnic group [27]. US Hispanics vary widely in terms of nativity and country of origin, population share, income, education level, English language orientation, and geographic mobility [28–32]. For example, the share of those with a bachelor’s degree or higher varies widely with 55% of Venezuelans, 43% of Argentines, 27% of Cubans, 19% of Puerto Ricans, 18% of Dominicans, 12% of Mexicans, and 10% of Guatemalans [28]. It has been suggested that health patterns vary by Hispanic subgroup due to the distinct cultural, socioeconomic, and political histories as well as settlement patterns of each group, all of which are hypothesized to affect health outcomes [33, 34]. Previous work has also found substantial differences in factors associated with health and health outcomes, such as social cohesion or the degree of solidarity/connectedness within a social community [35]. Importantly, there exist significant differences in mortality and other health outcomes across Hispanic subgroups [30]. These differences including obesity rates—which underlie multiple health issues among Hispanics—are much higher for Puerto Ricans and Mexicans than South Americans [34, 36, 37]. Disparate cultural and social characteristics, and differences in risk factors, mortality, morbidity, and healthcare access have been observed within the Hispanic grouping by nation of origin [12, 38].

Data availability constraints and historical convention have propagated the practice of examining health disparities for the Hispanic population in the aggregate, potentially masking heterogeneities across origin-nation and subgroups. As a result, despite the variation in cultural and social characteristics of this broad ethnic group, few studies provide a quantitative investigation of the healthcare use by Hispanic subgroups [39, 40]. We contribute to the literature by examining the relationship between Hispanic provider and patient concordance on healthcare utilization measures across Hispanic subgroups. An understanding of the heterogeneities within the Hispanic population would improve efforts to eradicate disparities in health outcomes and to promote preventative care [27, 41, 42].

## Methods

This study uses the Medical Expenditure Panel Survey—Household Component (MEPS HC), a subsample of households participating in the previous year’s National Health Interview Survey (NHIS) conducted by the National Center for Health Statistics (for more details see 3, 6). The survey consists of non-institutionalized civilians from the US population. About 30,000–35,000 respondents are surveyed annually [4]. The response rate ranges from 58 to 66% [4]. The study’s findings should not be generalized to individuals without a usual source of care provider.

This study limits data to adults who self-reported as Hispanic for years 2007 to 2017 and for whom complete data were available.^1^ Information pertaining to the race and ethnicity of the provider is reported by the participating households. Our sample includes 59,158 observations, including 43,958 (74.3%) Mexican respondents, 6264 (10.6%) Puerto Rican respondents, 3034 (5.1%) Cuban respondents, 2849 (4.8%) Dominican respondents, and 3053 (5.2%) respondents classified in the survey as Other Hispanics. About 46% of the respondents (26,924) were surveyed in English, 46% (27,002) were surveyed in Spanish, 8.9% (5226) were surveyed in both English and Spanish, and less than 1% (6) were surveyed in an “Other” language. The dataset consists of 33,379 (56%) foreign-born respondents. A total of 8% (4678) of cases in the sample involved Hispanic provider-patient concordance. The Hispanic provider-patient concordance does not disaggregate by categories.

## Variables

We include three standard measures of utilization from the MEPS HC data. The MEPS HC data does not include all three measures of utilization after 2017. The three measures are categorical (binary) outcome measures and include the probability of seeking preventive care, the probability of obtaining care for new health problem, and the probability of seeking continuing care for an ongoing health problem.

Similar to previous studies, covariates from five general categories were included: race and ethnicity concordance, non-health-related socioeconomic and demographic factors, health-related characteristics, provider communication characteristics, and provider location characteristics [3, 6, 38, 43]. Our non-health related socioeconomic and demographic covariates include immigration status (US-born citizens, those in the USA for less than 5 years, and those in the USA for greater than 5 years), age, sex, and marital status (married/single), education (less than a high school degree, high school degree, and college/advanced degree), family income (poor, low, middle, and high), US Census region (Northeast, Midwest, South, and West), and insurance status. Health-related characteristics include self-reported physical condition (Poor, Fair, Good, Very Good, and Excellent), any functional limitations, and self-reported chronic conditions (hypertension, diabetes, cholesterol, and heart diseases). The provider characteristics covariates include both location and convenience factors (ease in contacting by phone, weeknights and weekend office hours, and travel time is less than 30 min) as well as communication factors such as whether the provider speaks the person’s language and ratings of how well provider communicates with the patient (listens, explains, has respect for patient, and spends enough time with patient). We also include an interaction term between the Hispanic provider-patient concordance and the covariate indicating whether the patient was born in the USA.

Following previous studies, we estimate the multivariate non-linear probability models for each of the three dichotomous measures of health utilization using probit analyses [3, 6, 38]. The MEPS data is adjusted with survey weights to provide a nationally representative estimate. All models included the Hispanic patient-provider concordance, non-health related socioeconomic and demographics, health-related characteristics, and provider characteristics. Marginal effects are identified by the coefficients of the probit model and allow for the direct interpretation of the association between the probability of the utilization measure and the covariates. For example, the coefficient on the Hispanic patient-provider concordance is interpreted as the change in the probability of seeking care from a provider if that provider is also Hispanic. To estimate the Hispanic patient-provider concordance we defined a match as consisting of a situation when both provider and patient are identified as Hispanic. For example, a Hispanic patient-relationship would be one in which the individual identifies themselves as Hispanic and answers yes to the following question in the survey: “IS PROVIDER HISPANIC OR LATINO?” We also conducted the Wald Test in which the null hypothesis is that a set of parameters is equal. A rejection of the null hypothesis suggests that the coefficients are statistically different from one another. All analyses were carried out using StataSE17.

## Results

Table 1 presents the marginal effects of the multivariate non-linear probability models for each of the binary measures of utilization, “seeking preventative care,” “seeking care for a new problem,” and “seeking care for an ongoing problem” for the years 2007–2017. In general, Hispanic ethnic/ancestry group (Mexican, Cuban, Puerto Rican) did not show any significant marginal effects on care. Dominicans were more likely to seek care for an ongoing problem relative to the default group in the estimation. Namely, the findings suggest a lack of association for Hispanic ethnic/ancestry group and the likelihood to seek medical care for preventative care, new problems, or ongoing problems. Controlling for the covariates of race and ethnicity, non-health related socioeconomic and demographics, health-related characteristics, and provider characteristics, Hispanic patient-provider concordance was associated with statistically significant correlation with higher probabilities of seeking preventive care (coef=.211, *P*<.001), seeking care for a new problem (coef=.208, *P*<.001), and seeking care for an ongoing problem (coef=.208, *P*<.001), relative to Hispanic patients with a non-Hispanic provider. Patients 65 and older as well as women were more likely to seek medical care for preventive care (coef=0.054, *P*=.001 and coef=.029, *P*<.001, respectively), new medical problems (coef=0.037, *P*=.017 and coef=.034, *P*<.001, respectively), and ongoing medical problems (coef=0.046, *P*=.005 and coef=.025, *P*=.001, respectively). Relative to those born in the USA, foreign-born Hispanic patients were less likely to seek medical attention for preventive care irrespective of whether they lived in the country for less than 5 years (coef=−.156, *P*<.001) or more than 5 years (coef=−.166, *P*<.001). The results for foreign-born were similar for Hispanic patients seeking care for new problems (<5 years in US coef=−.101, *P*<.001; >5 years in US coef=−.064, *P*<.001) and ongoing problems (<5 years in US coef=−.155, *P*<.001; >5 years in US coef=−.070, *P*<.001). Relative to those with a college/advanced education, Hispanic patients without a high school degree (coef=−.029, *P*=.006) or those with only a high school degree (coef=−.021, *P*=.040) were less likely to seek preventive care. The provider characteristics that increase the likelihood of seeking medical care were as follows: whether it was easy to contact the provider by phone (coef=.471, *P*<0.001 for preventive care, coef=.471, *P*<.001 for new problem, and coef=.465, *P*<.001 for ongoing problem); has office hours at nights or over the weekends (coef=.181, *P*<0.001 for preventive care, coef=.203, *P*<.001 for new problem, and coef=.205, *P*<.001 for ongoing problem); and having a travel time less than 30 minutes (coef=.654, *P*<0.001 for preventive care, coef=.648, *P*<.001 for new problem, and coef=.647, *P*<.001 for ongoing problem). Likewise, there was a positive association between having a provider who speaks the patient’s language and medical care utilization (coef=.256, *P*<0.001 for preventive care, coef=.230, *P*<.001 for new problem, and coef=.269, *P*<.001 for ongoing problem).

**Table 1:** Marginal effects of the probability model of healthcare utilization, 2007–2017

	*Prob. of preventive care*				*Prob. of visit for new problem*				*Prob. of visit for ongoing problem*			
	Coef	95% CI		*P*	Coef	95% CI		*P*	Coef	95% CI		*P*
Race and ethnicity												
Other Hispanic	*Reference*				*Reference*				*Reference*			
Cuban	−.036	−.085	.012	.129	−.042	−.091	.006	.077	−.011	−.057	.036	.650
Dominican	.030	−.013	.073	.184	.016	−.030	.059	.475	.051	.009	.092	.023
Mexican	−.006	−.037	.025	.704	−.011	−.041	.020	.495	.006	−.025	.036	.718
Puerto Rican	.002	−.035	.039	.915	0.04	−.031	.041	.788	.005	−.032	.041	.789
Concordance												
Hispanic	.211	.194	.228	<.001	.208	.193	.224	<.001	.208	.189	.227	<.001
Age												
25–34	*Reference*				*Reference*				*Reference*			
35–44	.002	−.017	.021	.821	.008	−.011	.026	.401	.001	−.019	.020	.961
45–54	−.002	−.024	.019	.842	.011	−.009	.032	.284	.007	−.015	.028	.534
55–64	.031	.006	.057	.017	.003	−.023	.029	.834	.021	−.005	.047	.118
65+	.054	.025	.084	.001	.037	.008	.067	.017	.046	.015	.076	.005
Female	.029	.015	.043	<.001	.034	.020	.048	<.001	.025	.011	.039	.001
Married	.004	−.011	.019	.603	−.004	−.019	.010	.576	−.004	−.019	.011	.574
US status												
US born	*Reference*				*Reference*				*Reference*			
< 5 yr in the USA	−.156	−.195	−.116	<.001	−.101	−.180	−.101	<.001	−.155	−.194	−.117	<.001
> 5 yr in the USA	−.066	−.082	−.050	<.001	−.064	−.079	−.048	<.001	−.070	−.086	−.054	<.001
Family income												
High	*Reference*				*Reference*				*Reference*			
Poor	.002	−.022	.025	.893	−.018	−.042	.006	.136	−.025	−.049	−.000	.046
Low	−.009	−.034	.015	.447	−.032	−.057	−.007	.010	−.033	−.058	−.008	.009
Middle	−.012	−.035	.011	.297	−.034	−.057	−.011	.003	−.026	−.048	−.003	.027
Education												
College or adv. deg.	*Reference*				*Reference*				*Reference*			
No high school deg.	−.029	−.051	−.008	.006	−.019	−.040	.002	.068	−.012	−.033	.009	.262
High school deg.	−.021	−.040	−.001	.040	−.009	−.028	.010	.367	−.001	−.020	.019	.941
US census region												
Northeast	*Reference*				*Reference*				*Reference*			
Midwest	.003	−.030	.035	.875	−.042	−.076	−.008	.013	−.016	−.049	.017	.338
South	−.093	−.121	−.064	<.001	−.084	−.112	−.056	<.001	−.072	−.100	−.044	<.001
West	−.043	−.071	−.015	.003	−.044	−.072	−.017	.002	−.041	−.069	−.013	.004
Self-report health status												
Excellent	*Reference*				*Reference*				*Reference*			
Poor	−.025	−.092	.043	.456	.000	−.062	.063	.988	.048	−.013	.109	.140
Fair	.013	−.004	.030	.146	.018	.001	.035	.041	.024	.006	.041	.008
Good	.008	−.007	.022	.299	.006	−.008	.019	.437	.008	−.006	.023	.042
Very good	.008	−.017	.023	.277	.010	−.005	.024	.195	.001	−.014	.016	.893
Chronic condition												
Hypertension	.037	.018	.056	<.001	.028	.009	.046	.005	.054	.034	.072	<.001
Heart cond./disease	.018	−.009	.044	.0192	.009	−.017	.035	.494	.027	.000	.053	.051
Cholesterol	.054	.036	.072	<.001	.052	.035	.070	<.001	.051	.032	.069	<.001
Diabetes	.067	.044	.090	<.001	.039	.016	.062	.002	.059	.035	.083	<.001
Any functional limits	.068	.050	.087	<.001	.071	.053	.089	<.001	.075	.057	.094	<.001
Insurance	.017	−.001	.034	.059	.021	.004	.037	.019	.012	−.005	.030	.170
Convenience												
Phone contact	.471	.458	.483	<.001	.471	.459	.484	<.001	.465	.452	.477	<.001
Office hours	.181	.166	.195	<.001	.203	.189	.217	<.001	.205	.190	.220	<.001
< 30 m travel	.654	.643	.665	<.001	.648	.637	.659	<.001	.647	.636	.658	<.001
Communication												
Listens	.014	−.005	.032	.139	.019	.001	.037	.040	.022	.003	.040	.024
Explains	.035	.016	.053	<.001	.009	−.009	.027	.318	.021	.002	.040	.029
Respect	.003	−.016	.023	.732	.001	−.018	.020	.921	.007	−.013	.027	.488
Enough time	.004	−.013	.021	.646	.022	.006	.039	.007	.009	−.008	.025	.306
Prvdr. spks pat.’s lang	.256	.243	.269	<.001	.230	.217	.243	<.001	.269	.255	.047	<.001
Year fixed effects	Year				Yes				Yes			
Pseudo *R*^2^	0.806				0.816				0.789			
Number of obs.	59,158				59,158				59,158			

For brevity, Tables 2–3 display the disaggregated Hispanic categories and the Hispanic patient-provider concordance along with the interaction term for foreign-born. Table 2 presents the Hispanic patient-provider concordance disaggregated by Hispanic patient subgroups. The positive and statistically significant coefficients for all three health utilization measures across all Hispanic categories suggest that the Hispanic patient-provider concordance is associated with an increase in the likelihood of all Hispanics, irrespective of origin or culture, to seek medical care if the provider is Hispanic. However, a Wald test suggests that the association was not equal across the Hispanic subgroups. The association was lowest for Mexicans in preventive care (coef=.165, *P*<.001) and new problems (coef=.165, *P*<.001) and highest for Cubans in preventive care (coef=.256, *P*<.001) and ongoing problems (coef=.284, *P*<.001). It should be noted that the 95% confidence intervals for Mexicans in preventive care and Cubans in preventive care overlap. Table 3 shows that the results were robust to the interaction of the Hispanic patient-provider concordance for the Hispanic patient subgroups and being foreign-born.

**Table 2:** Disaggregated Hispanic categories and marginal effects of the probability model of healthcare utilization, 2007–2017

	*Prob. of preventive care*				*Prob. of visit for new problem*				*Prob. of visit for ongoing problem*			
	Coef	95% CI		*P*	Coef	95% CI		*P*	Coef	95% CI		*P*
Race and ethnicity												
Other Hispanic	*Reference*				*Reference*				*Reference*			
Cuban	−.073	−.127	−.019	.006	−.068	−.121	−.014	.009	−.059	−.112	−.006	.023
Dominican	.028	−.016	.071	.230	.007	−.038	.051	.776	.050	.008	.093	.026
Mexican	.001	−.030	.032	.950	−.005	−.035	.026	.765	.008	−.023	.040	.612
Puerto Rican	.002	−.035	.039	.956	.004	−.032	.040	.824	.007	−.030	.044	.713
Concordance												
Discordance	*Reference*				*Reference*				*Reference*			
Cuban	.256	.145	.267	<.001	.231	.218	.245	<.001	.284	.274	.294	<.001
Dominican	.224	.188	.259	<.001	.237	.227	.247	<.001	.196	.139	.254	<.001
Mexican	.165	.142	.189	<.001	.165	.143	187	<.001	.173	.148	.198	<.001
Puerto Rican	.230	.199	.261	<.001	.220	.196	.244	<.001	.168	.109	.226	<.001
Other Hispanic	.232	.197	.268	<.001	.216	.184	.249	<.001	.200	.133	.268	<.001
Year fixed effects	Year				Yes				Yes			
Pseudo *R*^2^	0.807				0.817				0.789			
Number of obs.	59,159				59,159				59,159

**Table 3:** Interaction of disaggregated Hispanic categories with foreign-born and marginal effects of the probability model of healthcare utilization, 2007–2017

	*Prob. of preventive care*				*Prob. of visit for new problem*				*Prob. of visit for ongoing problem*			
	Coef	95% CI		*P*	Coef	95% CI		*P*	Coef	95% CI		*P*
Race and ethnicity												
Other Hispanic	*Reference*				*Reference*				*Reference*			
Cuban	−.058	−.110	−.007	.020	−.058	−.110	−.007	.019	−.041	−.091	.009	.102
Dominican	.032	−.010	.075	.151	.011	−.033	.055	.618	.058	.016	.099	.010
Mexican	−.003	−.034	.028	.858	−.008	−.039	.022	.588	.009	−.022	.040	.584
Puerto Rican	.001	−.036	.037	.979	.004	−.032	.040	.839	.008	−.028	.045	.653
Interaction terms												
Discordance × For.	*Reference*				*Reference*				*Reference*			
Cuban × Foreign	.251	.237	.266	<.001	.230	.214	.245	<.001	.281	.269	.293	<.001
Dominican × Foreign	.201	.146	.256	<.001	.235	.221	.250	<.001	.154	.075	.233	.009
Mexican × Foreign	.140	.107	.174	<.001	.142	.111	.172	<.001	.143	.108	.178	<.001
Puerto Rican × For.	.236	.195	.277	<.001	.201	.149	.253	.001	.101	−.001	.204	.091
2003Other Hispanic × For.	.214	.134	.294	.017	.190	.102	.276	.031	.238	.159	.317	.009
Year fixed effects	Year				Year				Year			
Pseudo *R*^2^	0.805				0.815				0.788			
Number of obs.	59,158				59,158				59,158			

## Discussion

Our results add to a body of evidence supporting the hypothesis that patient-provider racial and ethnic concordance is associated with higher rates of healthcare utilization [4, 15] for Hispanic patients, contrary to previous findings that racial and ethnic concordance was not associated with Hispanic patients’ probability of utilizing health services [24]. Mixed and inconclusive findings in the literature may be due to differences in empirical specifications; differences in measures of patient satisfaction and healthcare utilization; and, as stressed in our study, aggregating across Hispanic subgroups. There are significant differences in risk factors, morbidity, mortality, access to care, and utilization of care observed among Hispanics by origin-country [38, 44]. Using an aggregated measure of the Hispanic demographic may contribute to mixed findings in previous studies which largely omits consideration of disaggregated subgroups by country of origin. The current work seeks to avoid this by examining association between patient-provider racial and ethnic concordance for specific Hispanic subgroups. By analyzing healthcare utilization for Hispanic subgroups, rather than in general, we were able to identify differences that would otherwise be hidden. While having a Hispanic provider was associated with a higher likelihood of seeking medical care for the Hispanic patients in the MEPS HC survey, the findings indicate that the association varies across Hispanic subcategories. For example, Cubans are least likely among the Hispanic subgroups to utilize healthcare in general, but most likely to seek care for preventive measures and ongoing problems if the provider is Hispanic. Our findings are consistent with previous studies which find evidence of differences in health behaviors, such as smoking and physical activity levels, [34, 45], dietary behaviors [46], and health status and outcomes [27, 47] across Hispanic subgroups.

Our results suggest the association between racial and ethnic concordance was stronger for Cuban, Dominican, and Puerto Rican patients compared to the reference category than it was for Mexican patients. Acculturation may explain some of the differences in utilization and the association of concordance demonstrated in this study. Previous work has exposed a link between acculturation and Hispanic patient satisfaction with medical care, with those who have spent a greater proportion of their lives in the USA, having reported higher levels of satisfaction with their medical care than less acculturated patients [15]. Acculturation may be an important factor in healthcare utilization given the variation of foreign-born across Hispanic subgroups with 56% for Cuban-origin Hispanics, 53% for Dominican-origin Hispanics, and 29% for Mexican-origin [48]. An implication of this heterogeneity is that it highlights the need for culturally competent care for recent immigrant groups, as lower levels of acculturation have a negative impact on the level of satisfaction with care [15]. Considering the findings establishing the association between patient satisfaction, racial and ethnic patient-provider concordance, and healthcare utilization [49], these subcategory differences should be taken into account when designing outreach programs.

## Conclusion and Limitations

In summary, racial disparities were observed in health utilization within Hispanic subgroups. While Hispanic patient-provider concordance is statistically significant in associating with healthcare utilization, the findings indicate that this association varies across Hispanic subpopulations. The observations suggest the importance of disaggregating Hispanic racial and ethnic categories into more similar cultural or origin groups. Linked with the existence of significant differences in mortality and other health outcomes across Hispanic subgroups, our results have implications for the design of community health promotion activities which should take these differences into account. Studies or community health programs which utilize generalized findings about Hispanic populations overlook differences across subgroups which may be crucial in promoting healthcare utilization. This suggests the need for more disaggregated data, targeted policy recommendations, enhanced cultural understanding, and increased representation in the healthcare workforce.

This study has several limitations. First, the data is unbalanced with more Mexican patients than any other Hispanic subgroups. It would be helpful to include greater disaggregation among Hispanic subgroups beyond Mexicans, Puerto Ricans, Cubans, and Dominicans to examine the variation more accurately across healthcare utilization measures. Second, we are unable to disaggregate the Hispanic provider to cultural or origin groups. Additionally, the study is limited as the survey does not provide racial identity of patients beyond “Hispanic.” Third, the cultural or ethnic identity of the provider reported in the MEPS data is perceived by the respondent, which may be inaccurately based on appearance and/or name. Fourth, the MEPS data is compiled from a self-reported survey which has limited external validation. Errors in self-reports may introduce bias in the data. Fifth, the study is also limited to the inclusion of respondents with a usual source of care (USC) for their providers. This limitation prevents generalization of the study to the population without USC [50]. Similarly, the findings in the study are limited to the population of non-institutionalized civilians in the survey. Lastly, we are unable to infer causal effects of racial and ethnic concordance as the MEPS data are cross-sectional. We thus limit our interpretations to associations only.

## Data Availability

All data are publicly available.

## Appendix A

MEPS survey questions about the past year for the respondent:

GO TO USC FOR PRVNTVE HLT CARE

1 Yes

2 No

GO TO USC FOR ONGOING HLTH PRB

1 Yes

2 No

GO TO USC FOR NEW HEALTH PROB

1 Yes

2 No

IS PROVIDER HISPANIC OR LATINO

1 Yes

2 No

RACE/ETHNICITY (EDITED/IMPUTED)

1 HISPANIC

2 NON-HISPANIC WHITE ONLY

3 NON-HISPANIC BLACK ONLY

4 NON-HISPANIC ASIAN ONLY

5 NON-HISPANIC OTHER RACE OR MULTIPLE RACE

HISPANIC ETHNICITY (EDITED/IMPUTED)

1 HISPANIC

2 NOT HISPANIC

## Footnotes

1 The period of the study is limited by the availability of data.

## Code Availability

Code available upon reasonable request after publication.
